# Measuring quality of health-care services: what is known and where are the gaps?

**DOI:** 10.2471/BLT.17.195099

**Published:** 2017-06-01

**Authors:** Margaret E Kruk, Edward Kelley, Shamsuzzoha B Syed, Finn Tarp, Tony Addison, Yoko Akachi

**Affiliations:** a Department of Global Health and Population, Harvard TH Chan School of Public Health, 677 Huntington Ave, Boston, MA 02115, United States of America.; b Department of Service Delivery and Safety, World Health Organization, Geneva, Switzerland.; c UNU-WIDER, Helsinki, Finland.; Correspondence to Margaret E Kruk (email: mkruk@hsph.harvard.edu).

The United Nations sustainable development goal (SDG) 3 seeks “to ensure healthy lives and promote well-being for all and at all ages”.^1^ To build health-care systems that were able to progress towards the millennium development goals, many countries had to extend delivery systems to increase coverage. They also greatly improved measurement of people’s contacts with the health system. However, with the reduction in disease burden due to specific infectious diseases and childhood illnesses, people tend to live longer, have multiple noncommunicable diseases and require more complex services. The focus on measuring access is not sufficient to capture whether people receive effective care; hence this month’s papers on measurement of quality of care in low- and middle-income countries.

In papers published online and in this issue, Akachi et al. explain why the quality of health-care services in low-and middle-income countries has been largely overlooked as an important contributor to health outcomes.^2^ Sharma et al. observe the management of childbirth at public and private hospitals in Uttar Pradesh, India and conclude that care provided to women and their newborns is of poor quality.^3^ Brenner et al. study the effects of a results-based financing scheme in Malawi and find improved equipment and supplies at health facilities but minimal effects on clinical performance.^4^ In Ethiopia, Canavan et al. measure the quality of intrapartum care in hospitals using data from medical chart reviews and direct observations.^5^

Knowlton et al. do a multinational survey of 120 hospitals and find that many lack the basic infrastructure needed to provide essential surgical care on a consistent basis.^6^ Lazzerini et al. find that in Kyrgyzstan – a setting with high rates of hospitalization, over-diagnosis and over-treatment – brief training and supportive supervision by paediatricians improve quality of paediatric care in hospitals.^7^ Examining variation in quality is one way to diagnose drivers of good or poor performance. Kruk et al. find that the quality of antenatal and paediatric care in seven African countries varies greatly and that this variation may result from the different approaches governments take in training providers and funding and organizing their health systems.^8^

Other articles in this issue present innovations in measures and instruments to assess quality of health-care services. Bedoya et al. document compliance with infection prevention and control measures during outpatient visits in Kenya.^9^ Wang et al. show how medical malpractice litigation records can be used as a source of data to assess patients’ experience and their health outcomes in China.^10^ Madaj et al. assess the validity of the World Health Organization’s indicators for quality of care around the time of birth.^11^

Despite the wide range of research presented in this issue, several aspects of health-care quality are not addressed. Most studies are limited to a few health facilities and thus are not generalizable to each country as a whole. Authors acknowledge this limitation. No studies examine patients’ perspectives of quality, patient-reported experiences, or associations between quality measures and health outcomes. While Hanson et al. discuss community-based approaches for newborn health,^12^ the role of community engagement and empowerment in driving demand for higher quality care elsewhere is left relatively untouched. It’s known that measurement and quality-improvement programmes have major resource implications, yet there is limited research that addresses costs, cost–effectiveness and efficiency of such efforts.

The articles in this issue provide a glimpse of current research on quality of health-care services in low- and middle-income countries. The authors make a strong case for the need for governments to both improve health-care quality and to be able to measure the effects of such improvements. Development partners can contribute to this work by developing and validating measurement standards, data collection tools and supporting evaluation research.

As a next step, quality of care should be integrated into existing policy dialogues. National quality policies and strategies need to reflect the trade-offs between the ability to measure, and the ability to deliver, higher-quality health services. Global indicators for quality, integration and people-centredness of health services and a framework for measurement in these areas will be proposed to the 2018 World Health Assembly to aid countries in their own monitoring efforts. Several global initiatives are also contributing to this work, including a Global Report on Quality jointly being produced by the Organisation for Economic Co-operation and Development, the World Health Organization and the World Bank, the Lancet Global Health Commission on High Quality Health Systems in the SDG era,^13^ Countdown to 2030,^14^ and a range of country quality networks on distinct subject areas, including maternal, newborn and child care. Better health is unlikely without better health-care quality, and improving health-care quality demands measurement that is accurate and usable by countries.
